# Nanodiamond enhances immune responses in mice against recombinant HA/H7N9 protein

**DOI:** 10.1186/s12951-017-0305-2

**Published:** 2017-10-05

**Authors:** Ngoc Bich Pham, Thuong Thi Ho, Giang Thu Nguyen, Thuy Thi Le, Ngoc Thu Le, Huan-Cheng Chang, Minh Dinh Pham, Udo Conrad, Ha Hoang Chu

**Affiliations:** 1 Institute of Biotechnology, Vietnam Academy of Science and Technology, Ha Noi, Vietnam; 2 Graduate University of Science and Technology, Vietnam Academy of Science and Technology, Ha Noi, Vietnam; 3 Institute of Atomic and Molecular Sciences, Academia Sinica, Taipei, 10617 Taiwan, ROC; 4 Leibniz Institute of Plant Genetics and Crop Plant Research, Gatersleben, Germany

**Keywords:** Nanodiamond, Recombinant H7 haemagglutinin, Influenza virus A/H7N9, Synthetic virus-like particles, Plant-derived protein

## Abstract

**Background:**

The continuing spread of the newly emerged H7N9 virus among poultry in China, as well as the possibility of human-to-human transmission, has attracted numerous efforts to develop an effective vaccine against H7N9. The use of nanoparticles in vaccinology is inspired by the fact that most pathogens have a dimension within the nano-size range and therefore can be processed efficiently by the immune system, which leads to a potent immune response. Herein, we report a facile approach to increase antigen size to achieve not only fast but also effective responses against the recombinant HA/H7N9 protein via a simple conjugation of the protein onto the surface of nanodiamond particles.

**Results:**

In this study, trimeric Haemagglutinin (H7) that is transiently expressed in *N. benthamiana* was purified using affinity chromatography, and its trimeric state was revealed successfully by the cross-linking reaction. The trimeric H7 solution was subsequently mixed with a nanodiamond suspension in different ratios. The successful conjugation of the trimeric H7 onto the surface of nanodiamond particles was demonstrated by the changes in size and Zeta-potential of the particles before and after protein coating, Sodium dodecyl sulfate polyacrylamide gel electrophoresis (SDS-PAGE), and Western-blot analysis. Next, biofunction of the protein-nanodiamond conjugates was screened using a haemagglutination assay. A mixture containing 5 µg of trimeric H7 and 60 µg of nanodiamond corresponds to a ratio of 1:12 (w/w) of agglutinated chicken red blood cells at HA titer of 1024, which is 512-fold higher than the HA titer of free trimeric H7. After the 2nd and 3rd immunization in mice, ELISA and Western blot analyses demonstrated that the physical mixture of trimeric H7 protein and nanodiamond (1:12, w/w) elicited statistically significant stronger H7-specific-IgG response demonstrated by higher amounts of H7N9-specific IgG (over 15.4-fold with P < 0.05 after the second immunization).

**Conclusions:**

These results indicated a potential effect inherent to nanodiamond towards modulating immune systems, which should be further evaluated and broadly applied in nanovaccine development.

## Background

Vaccination is one of the most effective and cost-beneficial interventions that prevent mortality and reduce morbidity caused by pathogens such as influenza viruses [22]. To improve host immune responses against continuing and emerging viral threats, vaccine technology continues to put great efforts to integrating virus-like features in vaccine formulations. Recent studies have illustrated that vaccines that preserve virus-like features showed better capability in eliciting immune responses in comparison with traditional vaccine formulations [1, 6, 10, 16].

In recent years, nanoparticles have been considered as an interesting component for experimental vaccine formulations [32]. The use of nanoparticles in vaccinology is inspired by the fact that most pathogens have dimensions in the nano-size range [31], and therefore can be processed efficiently by the immune system, which leads to a potent immune response. Therefore, nanoparticles are being exploited to elicit desired immune responses for both prophylactic and therapeutic effects. They are utilized either as a delivery system to enhance antigen processing or to protect antigen from premature degradation and/or as an immune stimulant to trigger immune responses [26]. Nanotechnology allows customization of the nanoparticle properties, such as size, shape and surface charge, to meet application requirements, which results in a significant variety of nanoparticles.

Nanodiamond (ND) is a carbon nanomaterial that has been applied in many fields of research [8, 14, 17, 18]. With excellent mechanical and optical properties, high surface areas, tunable surface structures and biocompatibility, nanomaterials are well suited to biomedical applications. Previous studies have shown that strong acid-oxidized NDs have a remarkably high affinity for proteins [2, 11]. Thus, proteins and NDs can form stable conjugates easily and effectively in different buffers via physical absorption. It is also unique that both soluble proteins and native membrane proteins solubilized in detergent micelles can be easily conjugated onto the surface of NDs, likely due to the intrinsic hydrophobicity of the carbon-based nanomaterial [17, 18]. This characteristic allows a facile and effective loading of a high amount of protein molecules on their surface for bio-medical applications. For ~ 100 nm NDs, a 20–30-µg weight of nanoparticles can carry a 1 µg dried weight of protein [2, 17].

Influenza viruses are serious global pathogens and are deemed to be one of the biggest threats to human and animal health. Avian influenza A (H7N9) virus emerged as a new human pathogen in China in spring of 2013. By February 2015, 571 human H7 infected cases have been reported including 212 deaths [29]. H7N9 viruses, along with many other emerging influenza subtypes, challenge scientists to develop faster and more efficacious vaccines. Haemagglutinin (HA), which is the major protein of influenza H7N9 virus envelope, contains virus-neutralizing epitopes and is included in all currently approved human influenza vaccines as well as in majority of experimental vaccines [5]. Native influenza HA protein is polymerized as a trimer. It seems that the recombinant HA fragments themselves cannot easily form a functional when expressing in different systems without motif fusion.

The recombinant subunit vaccine technology is a promising approach for developing novel influenza vaccines due to the advantages in safety and manufacturing. These vaccines have been shown to be efficacious against influenza over attenuated or inactivated vaccines [23, 24]. Some recent studies have shown that the H7N9 recombinant virus-like particle (VLP) vaccine induced protective immunity against the H7N9 virus in model animals [9, 12, 23]. However, production of these recombinant VLP vaccines is time-consuming. Plants have shown to be one of the most promising alternative pharmaceutical production platforms that are robust, scalable, low-cost and safe. A rapid H7-VLP vaccine development (19 days) from *Nicotiana benthamiana* is performed using an agro-infiltration-elicited protective immune response against the H7N9 influenza virus [21].

In the context of this study, an H7 fused GCN4-pII motif trimer (designated as H7) was physically mixed with NDs in an optimized ratio to generate oligomeric H7 as a synthetic H7-VLP formation. Trimeric H7 was expressed in *N. benthamiana*, purified via affinity chromatography (IMAC), characterized via a cross-linking reaction, and then incubated with a mixture of 50–500 nm NDs with various ratios. The optimal mixture of trimeric H7 and NDs (designated as H7:ND) was screened using the Western blot and haemagglutination assays that are based on the binding capacity of trimeric H7 on the surface of NDs and the ability of trimeric H7 to agglutinate chicken red blood cells (RBCs), respectively. The successful conjugation of trimeric H7 onto the surface of NDs was analysed using a particle analyser, SDS-PAGE, and Western blot. The trimeric H7 protein, NDs and the optimal mixture of H7:ND were injected in mice. Then, the immunogenicity induced by the optimal mixture of H7:ND in mice was compared to that of trimeric H7 as well as NDs only according to ELISA and Western blot.

## Methods

### Production of the recombinant trimeric H7 protein

#### Construction of the plant expression vector

**Fig. 1:**
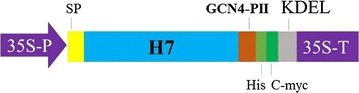
Construct for expression of trimeric Haemagglutinin in plants. H7 was expressed in tobacco leaves under the control of the CaMV 35S promoter. H7 was fused with trimeric motif GCN4-pII to form H7-pII. Recombinant H7 contained His and c-myc tags for affinity chromatography purification and Western blotting, respectively. The LeB4 signal peptide and KDEL motif were used to ensure ER retention. 35S-P: CaMV 35 S promoter; SP: legumin B4 signal peptide; H7: haemagglutinin A/H7N9; GCN4-pII: trimeric motif; His: His-tag; 35S-T: CaMV 35S terminator

The DNA sequence that encodes amino acids (aa) 2–560 of Haemagglutinin subtype 7 (H7) of Influenza Antigen A/Anhui/1/2013 (UniProt Accession A0A0K1LFL0) was optimized for expression in tobacco plants, synthesized commercially (GeneCust Europe, Luxembourg) and provided in pUC57 vectors (designated as pUC57-H7). The H5 gene in pRTRA-35S-LeB4SP-H5-pII-His-cmyc-KDEL [19] was replaced with the synthesized DNA sequence that encodes ectodomain H7 (aa 17–520) to generate expression cassettes (pRTRA-35S-LeB4SP-H7-pII-His-cmyc-KDEL) under the control of the CaMV 35S promoter. The expression cassette in the pRTRA vector (shown in Fig. 1) was sub-cloned via the Hind*III* cleavage into the shuttle vector pCB301-Kan. The pCB301-H7-pII shuttle vectors were introduced into the *Agrobacterium* pGV2206 strain.

#### Transient expression

*Agrobacterium* infiltration for expressing recombinant proteins was described by Phan et al. [20]. Bacteria that harbour the shuttle vectors pCB301-H7-pII and HcPro vector were suspended in an infiltration buffer [10 mM 2-(N-morpholino)ethanesulfonic acid (MES), 10 mM MgSO_4_, pH 5.6]. *N. benthamiana* plants (6–8 weeks old) were infiltrated by completely submerging each plant in an *Agrobacterium*-containing cup standing inside a desiccator and applying a partial vacuum for 2 min. Next, the plants were kept in a greenhouse at 21 °C, 16 h of light/8 h of dark for 6 days, and then leaf samples were harvested and stored at − 80 °C.

#### Protein purification via immobilized metal affinity chromatography (IMAC)

Six days after vacuum infiltration of *Agrobacterium*, the leaf samples were harvested, treated in liquid nitrogen and homogenized using a commercial blender. Total protein was extracted in a Tris buffer (50 mM Tris–HCl, pH 8.0). The extract was clarified via centrifugation twice at 75,465×*g*, 30 min, 4 °C. The clear extract was mixed with a Ni–NTA agarose resin that was previously washed twice with water and once with a Tris buffer. After mixing at 4 °C for 30 min, the mixture was applied to a chromatography column. Thereafter, the column was washed two times with a washing buffer (50 mM NaH_2_PO_4_, 300 mM NaCl, 30 mM imidazole, pH 8.0). Then, recombinant protein was eluted from the column with an elution buffer (50 mM NaH_2_PO_4_, 300 mM NaCl, 250 mM imidazole, pH 8.0), put into dialysis bags, concentrated in PEG 6000, and dialyzed against PBS (137 mM NaCl, 2.7 mM KCl, 10 mM Na_2_HPO_4_, 1.8 mM KH_2_PO_4_, pH 7.4) at 4 °C overnight.

#### Cross-linking reaction

To determine the multimeric state of the purified H7-pII protein, a cross-linking reaction was performed following the method described by Weldon et al. [28]. Briefly, 1 µg of recombinant proteins was mixed with Bis[sulfosuccinimidyl]suberate (BS3) to a 5 mM final concentration and incubated for 30 min at room temperature. The crosslinking reaction was stopped by adding 1 M Tris–HCl pH 8.0 to a final concentration of 50 mM and incubated for 15 min at room temperature. After cross-linking, the proteins were separated on a 4–10% SDS-PAGE under reducing conditions, blotted and analysed via Western blot using the above mentioned anti-c-myc monoclonal antibody.

### Production of surface-oxidized NDs

Diamond powder (Diamond Innovations, USA) was surface-functionalized with oxygen containing groups via a harsh treatment in a strong oxidative acid mixture (3 volume of H_2_SO_4_: 1 volume of HNO_3_) under microwave heating (~ 100 °C) as described in a previous publication [13]. The treatment is conducted in a microwave reactor (100 W, Model Discover, CEM) for 3 h. At the end of the microwave heating, precaution was carefully taken to ensure that the residual strong acids are diluted prior collection of the NDs. For safe operation, the microwave reactor is placed in a chemical fume-hood to protect the operator from NO_2_ exposure.

### Synthesis of the H7–ND complexes

Oxidative ND particles are first thoroughly re-suspended in DI-H_2_O with the assistance of sonication at the concentration of 10 mg/mL. To find the best conditions for maintaining the activity of protein samples as well as to obtain the good re-suspending nanoconjugates after protein coatings, various H7:ND ratios (w/w) of 1:1, 1:3, 1:7, 1:9, 1:12, and 1:15 were tested in different buffers such as 1 × PBS and DI-H_2_O. Sonication for 1 h was applied during coating.

### H7:ND characterization

#### Size and Zeta-potential measurements

Size distributions and Zeta-potentials of NDs before and after protein coatings were measured using a particle analyser (Delsa^@^NanoC, Backman Coulter, USA). For size distribution and Zeta potential measurements, bare-NDs and protein-coated FNDs are re-suspended in DI-H_2_O at the concentration of 50 µg/mL. To evaluate the aggregating tendency of the protein-coated ND complexes in salt buffers, the samples were re-suspended in 1 × PBS, followed by particle size measurements.

#### SDS-PAGE and Western blot of protein-coated NDs

The protein-coated ND particles were first washed twice with deionized water to remove loosely bound moieties, such as salts or excessive protein molecules, before elution with 20 µL of an SDS-PAGE loading buffer (3% SDS, 1.5% DTT, 0.1% bromophenol blue, 0.5 M Tris–HCl pH 6.8, 10% glycerol). Only the supernatants containing proteins were loaded onto the gels. Casting and running of protein gels was performed according to Laemmli using BioRad’s Miniprotean II. Western blots were performed following the protocol described by Gahrtz et al. [4]. ImageJ was then used to analyse the Western blot result based on the intensity of the H7 protein band.

#### Transmission electron microscopy (TEM) analysis

The shape and surface properties of surface-oxidized NDs, H7:ND complexes were further characterized via TEM images. ND and H7:ND complexes were re-suspended in DI-H_2_O at a concentration of 1 mg/mL. Then, the particles in solution are coated onto the TEM plate for image acquisition.

### Haemagglutination assay

Haemagglutination assays were performed following the World Organization for Animal Health recommendation [30]. In detail, 50 µL of PBS were added into all wells of a plastic V-bottom microtiter plate. Next, 50 µL of antigen was added into the first well of a plastic V-bottom microtiter plate. A 2-fold serial dilution was made across the entire row. In total, 50 µL of the last dilution was discarded. Furthermore, 50 µl of 1% chicken RBCs was added to each well. After incubating the plates at 25 °C for 30 min, the results were read. The endpoint dilution that causes complete haemagglutination was defined as one haemagglutination unit (HAU).

### Mouse experiment

The 6–8-week-old female BALB/C mice (five per group) were immunized using subcutaneous route on a schedule of 1, 14, 28 days with 5 µg of purified H7-pII trimer (group 1), a mixture of 5 µg of purified H7-pII trimer and 60 µg of NDs [at a ratio of 1:12 (w/w, group 2]. In the control group, mice were injected with a mixture of PBS and 60 µg of NDs (group 3). The antigens were formulated with complete Freund’s adjuvant in the first immunization and with incomplete Freund’s adjuvant in the booster immunizations. One week after the 2nd and 3rd immunization, the mice were bled via the retro-orbital sinus. Mouse sera were collected individually for ELISA tests and Western blot.

#### ELISA

For testing mouse sera, microtiter plates (ImmunoPlate Maxisorp, Nalgen Nunc International, Roskilde, Denmark) were coated with 100 µL of 50 ng H7 (Influenza Antigen A/Anhui/1/2013 (H7N9), NIBSC) in PBS (100 mM NaCl, 32 mM Na_2_HPO_4_, 17 mM Na_2_HPO_4_, pH 7.2) and incubated overnight. After blocking with 3% (w/v) bovine serum albumin (BSA), 0.05% (v/v) Tween20 in PBS (PBST) for 2 h, 100 µL of the specific mouse sera dilution [5000 times in 1% (w/v) BSA in PBST] and an anti H7N9 haemagglutinin/HA monoclonal mouse antibody (SinoBiological InC.) at concentrations of 0.5, 0.75, 1, 1.25, 2.5, 5, 12.5, 25, 50, 100, 150 μg/mL was applied on each ELISA plate and incubated at 25 °C for 1 h. Each serum dilution was measured in triplicate. The plates were washed 5 times with PBST, followed by the addition of 100 µl of a goat anti-mouse IgG conjugate horseradish peroxidase (HRP) dilution (2000 times in 1% (w/v) BSA in PBST and then incubated at 25 °C for 1 h. The enzymatic substrate, 1-Step™ Ultra TMB-ELISA Substrate Solution (Thermo Fisher Scientific, Lithuania) was added. After 20 min of incubation, the reaction was stopped via 1 M HCl. All mouse sera were applied at the same ELISA conditions. The absorbance signal was measured at 450 nm. The ELISA values of all mouse sera were normalised by use of a monoclonal mouse standard H7N9 haemagglutinin/HA antibody (SinoBiological Inc.). A standard curve was built using the OD450 values corresponding to different concentrations of this antibody. Control values for BSA at the solid phase were subtracted. The amounts of specific anti H7N9 haemagglutinin/HA antibodies in the sera were calculated using this standard curve.

#### SDS-PAGE and Western blot

Proteins were separated in a 12% SDS-PAGE gel and transferred to a PVDF membrane (Millipore) via a semidry Fast Blotter (Thermo Scientific). After blocking with a 5% (w/v) fat-free milk powder dissolved in a PBS buffer (137 mM NaCl, 2.7 mM KCl, 10 mM Na_2_HPO_4_, 1.8 mM KH_2_PO_4_, pH 7.4), the membrane was incubated for 2 h at room temperature with the addition of a 1:50 dilution of monoclonal anti-c-myc antibody and later with HRP conjugated goat anti-mouse IgG secondary antibody at a 1:2000 dilution.

To detect the H7-specific IgG mouse antibodies, each 100 ng of purified H7 (Influenza Antigen A/Anhui/1/2013 (H7N9), NIBSC) was loaded on 7 lanes of one SDS-PAGE gel. The proteins were separated according to their molecular weight and transferred to a PVDF membrane (Millipore) via a semidry Fast Blotter (Thermo Fisher Scientific, USA). After 2 h blocking with a 5% (w/v) fat-free milk powder dissolved in a PBS buffer (137 mM NaCl, 2.7 mM KCl, 10 mM Na_2_HPO_4_, 1.8 mM KH_2_PO_4_, pH 7.4), 7 lanes of the membrane were separated via cutting. Then, the single stripes were incubated for 2 h at room temperature with a 1:500 dilution of mixture of five mice sera of each group (group 1, group 2 and group 3). Anti-mouse H7N9 Haemagglutinin/HA antibody (SinoBiological InC., China) was used as a positive control (P). Next, the membranes were incubated for 1 h at room temperature with the addition of a 1∶2000 dilution of an HRP conjugated goat anti-mouse IgG secondary antibody.

Specific signals were visualized by incubating membranes in dark with 3,3-diaminobenzidine (DAB, Thermo Scientific Pierce) in 0.05 M Tris–HCl and 0.04% hydrogen peroxide for 10 min.

### Statistical analysis

Statistical analyses for ELISA test were performed using t-test in the Sigma Plot software. The mean difference between sample data was compared and is presented as X ± Standard deviation (SD). P values that are less than 0.05 were defined as significant difference.

## Results

### Production and characterization of H7 Haemagglutinin

#### Expression of influenza H7 Haemagglutinin in plants

To efficiently express H7 in plant cells, the sequence that encodes ectodomain (aa 17–520) of the Influenza Antigen A/Anhui/1/2013 strain was synthesized with optimized codons and cloned in an expression cassette (Fig. 1). The H7 protein was recombinantly expressed as a fusion with a trimeric motif (GCN4-pII) under the control of CaMV 35S. The recombinant H7 protein contained the c-myc tag for downstream detection via Western blot, a His tag for purification via IMAC and the KDEL motif at the C-terminal end to retain protein in the endoplasmic reticulum (ER).

**Fig. 2:**
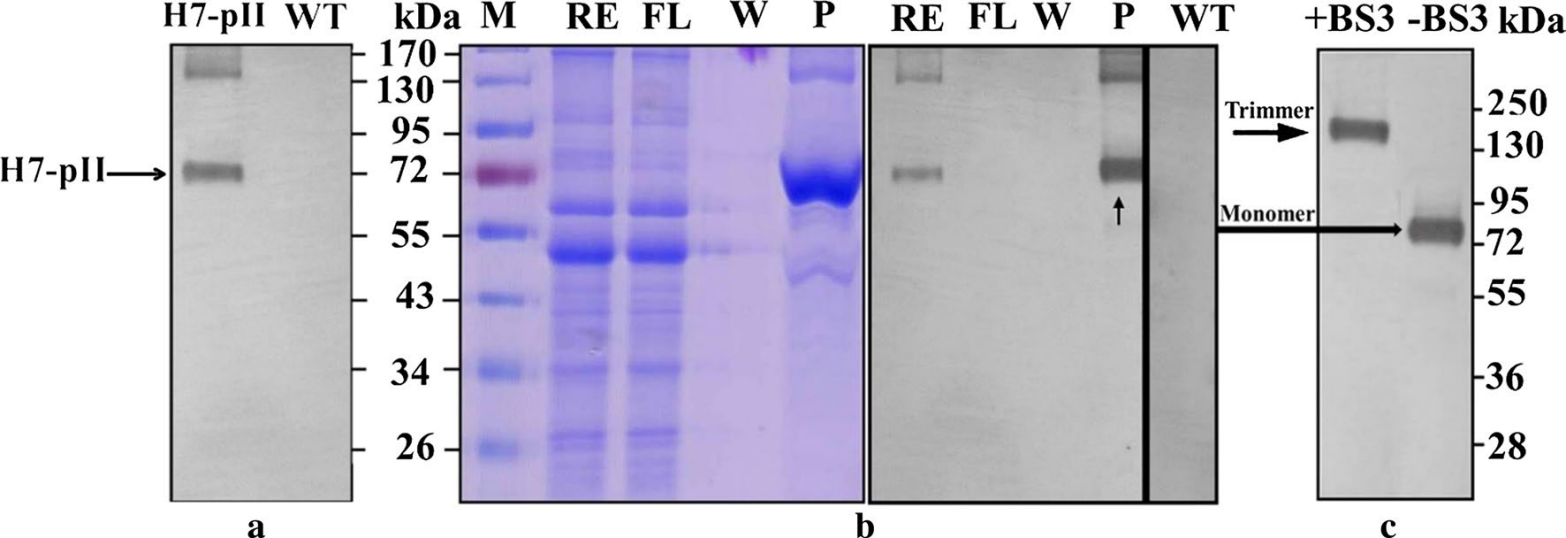
Purification and characterizations of recombinant trimeric H7 expressed in *N. benthamiana* leaves. **a** Recombinant H7-pII produced from agro-infiltrated tobacco leaves and detected by Western blot: H7-pII: 20 µg of total soluble protein extracts from tobacco leaves; Wt: wild type used as the negative control. **b** Purification of H7-pII from total soluble protein extracts using IMAC: *RE* raw extract, *FT* flow through, *W* wash fraction, *P* purified fraction in SDS-PAGE (Left) and H7-pII immunological detection via anti-c-myc monoclonal antibody (Right); *WT* wild type *N. benthamiana*; *M* protein marker (Right). **c** Detection of oligomeric states of H7-pII via the cross-linking reaction: (−) and (+) BS3: indicate a cross-linker bis(sulfosuccinimidyl)substrate (BS3) with 0 and 5 mM final concentration, respectively. The resulting products were separated via the 4–10% gradient SDS-PAGE under reducing conditions, blotted and detected via anti-c-myc monoclonal antibody

The functionality of the binary vector containing H7-pII was validated via transient expression in *N. benthamiana* leaves, determined via SDS-PAGE under reducing conditions, and Western blot using an anti-c-myc monoclonal antibody (Fig. 2a). Western blot analysis indicates that recombinant H7-pII was successfully expressed in plants. The apparent molecular weight shown in Fig. 2a is higher than the expected sizes predicted from the polypeptide sequence of H7. This can be explained by the fact that glycosylation influences the running behaviour during the electrophoretic separation.

#### Purification and characterizations of recombinant H7 expressed in *N. benthamiana* leaves

To obtain recombinant H7 for further experiments, the H7-pII protein containing a C-terminal 6 × histidine tag was purified from plant leaves via IMAC. The samples from each step of the purification procedure were collected and analysed via SDS-PAGE and Western blot using a monoclonal-anti c-myc antibody (Fig. 2b). Western blot analysis reveals that one band corresponds to H7-pII in the elution fraction. These results indicate that the H7-pII protein was highly enriched from tobacco plant leaves and successfully purified.

After purification of the H7-pII protein, the oligomeric state of this protein was determined via a cross-linking reaction using a BS3 chemical, a water-soluble and homobifunctional cross-linker that reacts with primary amines of proteins to form stable amide bonds. When oligomeric proteins were exposed to BS3, cross-links between each subunit of the multimeric proteins are formed. This is a direct evidence for their close proximity. Following cross-linking, reaction products were separated on a gradient of 4–10% SDS-PAGE under reducing conditions, blotted and immune detected using an anti-c-myc monoclonal antibody. The immunoblot results in Fig. 2c revealed a band with a molecular weight of approximately 220 kDa after H7-pII was cross-linked via BS3. This result indicates that H7-pII is an exclusively trimeric protein.

### Production and characterization of surface-oxidized NDs

**Fig. 3:**
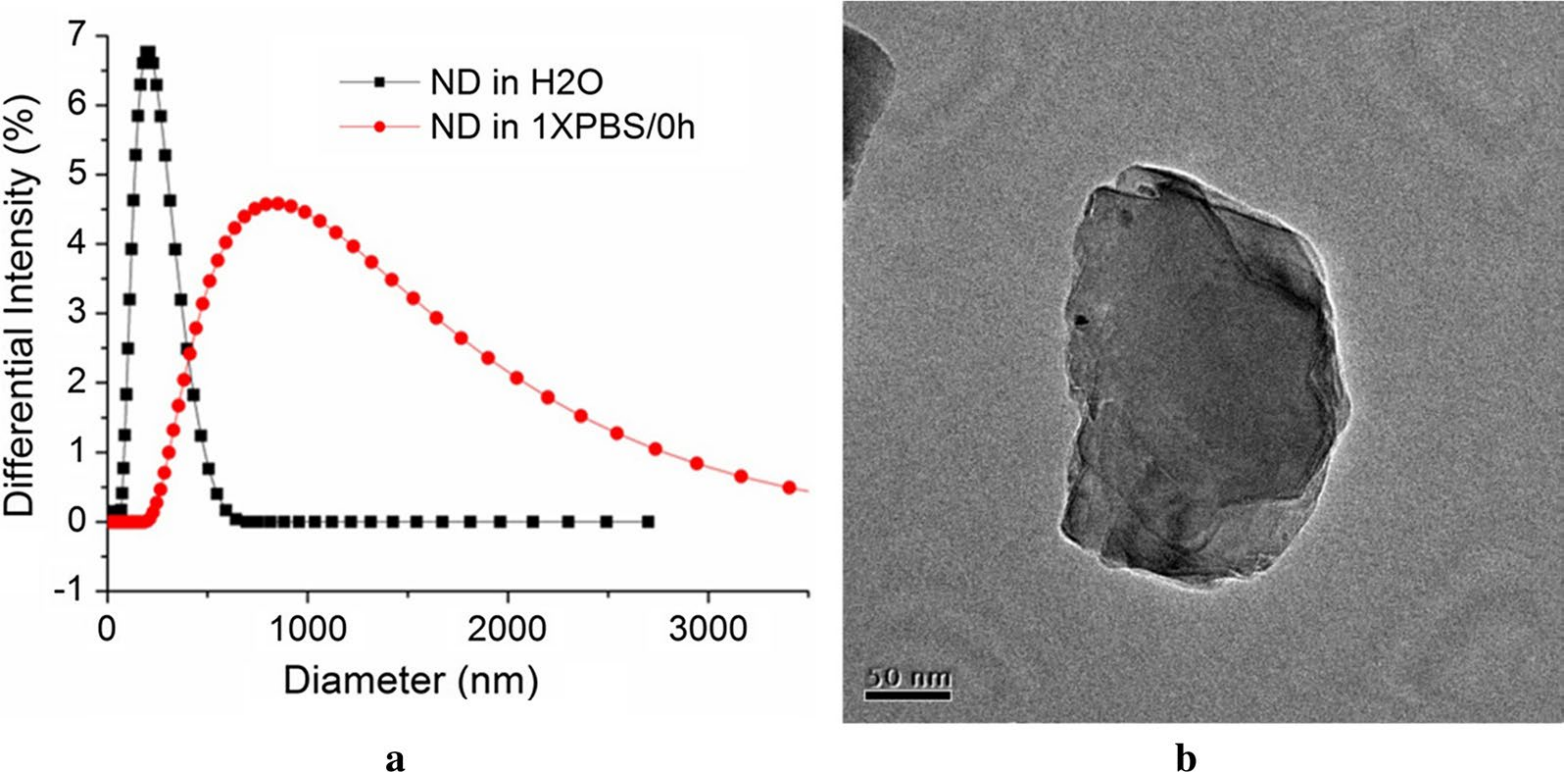
Preparation and characterization of nanodiamonds. **a** Size distribution of re-suspended nanodiamonds in H_2_O and 1 × PBS; **b** TEM image of nanodiamond resuspension in H_2_O

The size distribution and TEM analysis of NDs used in the present experiment are shown in Fig. 3. It is clearly observed that we have a mixture of ND particles with a wide range of sizes from ~ 50 to ~ 500 nm in diameter (Fig. 3a). The size distribution of NDs in H_2_O suggested that more than two particles can from aggregates, and there may be a variation in size between different particles. Indeed, the TEM analysis illustrated on Fig. 3b shows both a single ND and an aggregate of 2 ND particles. In addition, we can see from the TEM images that the NDs are not round, and individual particles are very different in shape. It is also noted from Fig. 3a, that NDs can form a very bad aggregation in salt solutions such as 1 × PBS.

In the study, we used NDs that were oxidized in a mixture of strong acids including HNO_3_ and H_2_SO_4_ at a volume ratio of 1:3. After the harsh acid treatment, NDs are decorated with oxygen-containing functional groups, such as carboxylic acids, and thus have a negatively charged surface.

### Synthesis and characterization of the H7:ND complexes

#### Synthesis and physical characterization of the H7:ND complexes

**Fig. 4:**
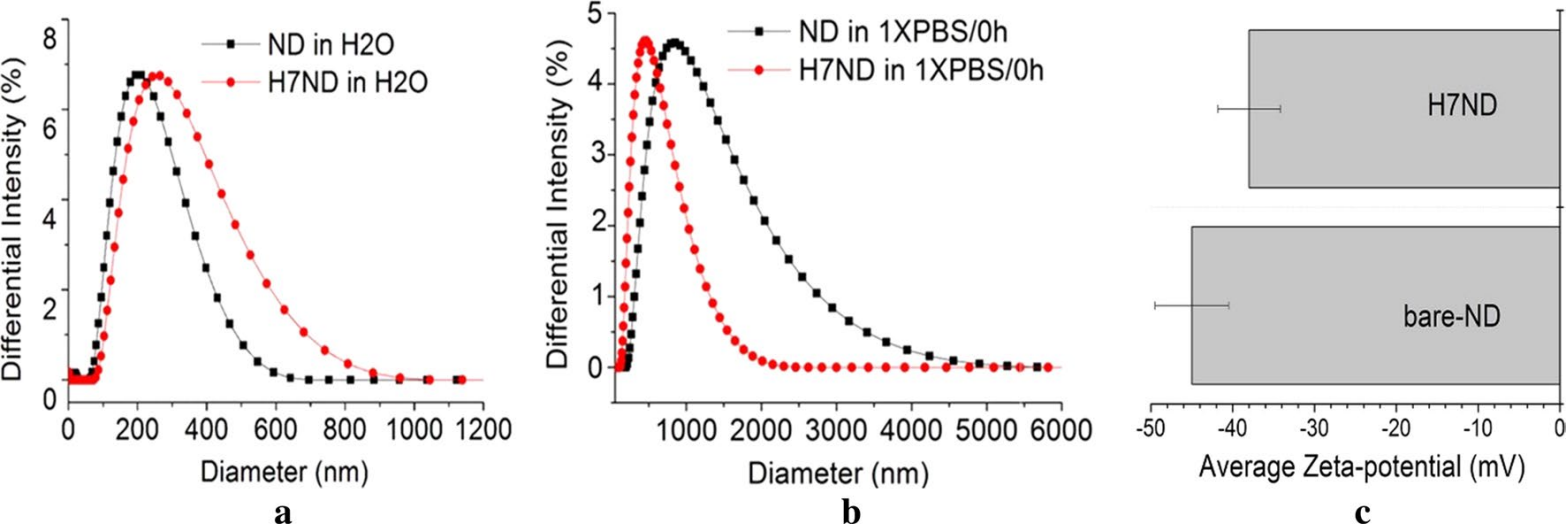
Synthesis and physical characterization of H7-ND conjugates. The re-suspension of nanodiamond particles before protein coating (ND) and after coating with trimeric H7 protein (H7:ND): **a** in H_2_O; **b** in 1× PBS; **c** Zeta-potential of H7:ND and ND

The H7:ND complexes were synthesized via a simple mixing of protein solution and ND suspension for 1 h under sonication. Different Protein/ND ratios (w/w) (1:1, 1:3, 1:5, 1:7, 1:9, 1:12 and 1:15) were tested to find the best synthetic condition. Figure 4 shows the changes in the size distribution of ND before and after coating with trimeric H7 protein at a 1:1 (w/w) ratio. The protein-coated ND samples were re-suspended in both DI-H_2_O and 1 × PBS for the particle size measurements. It can be clearly seen from Fig. 4a, b that ND particles increase in size to approximately 80 nm in diameter after protein coating. These results indicated that a significant amount of protein was conjugated onto the surface of ND particles. Comparing the size distribution of H7:ND and ND in 1 × PBS also revealed that protein coating helps preventing fast aggregation of the nanoparticles in a physiological condition.

To further analyse the change in surface chemistry of ND after H7 coating, we measured Zeta-potential of NDs in comparison with the H7:ND complexes. We found that both types of particles have negatively charged surfaces. Furthermore, the change of Zeta-potential is from ~ − 45 mV for ND to ~ − 38 mV for H7:ND (Fig. 4c).

### Characterization and bio-functional screening of the optimal mixture of purified trimeric H7 and nanodiamonds

**Fig. 5:**
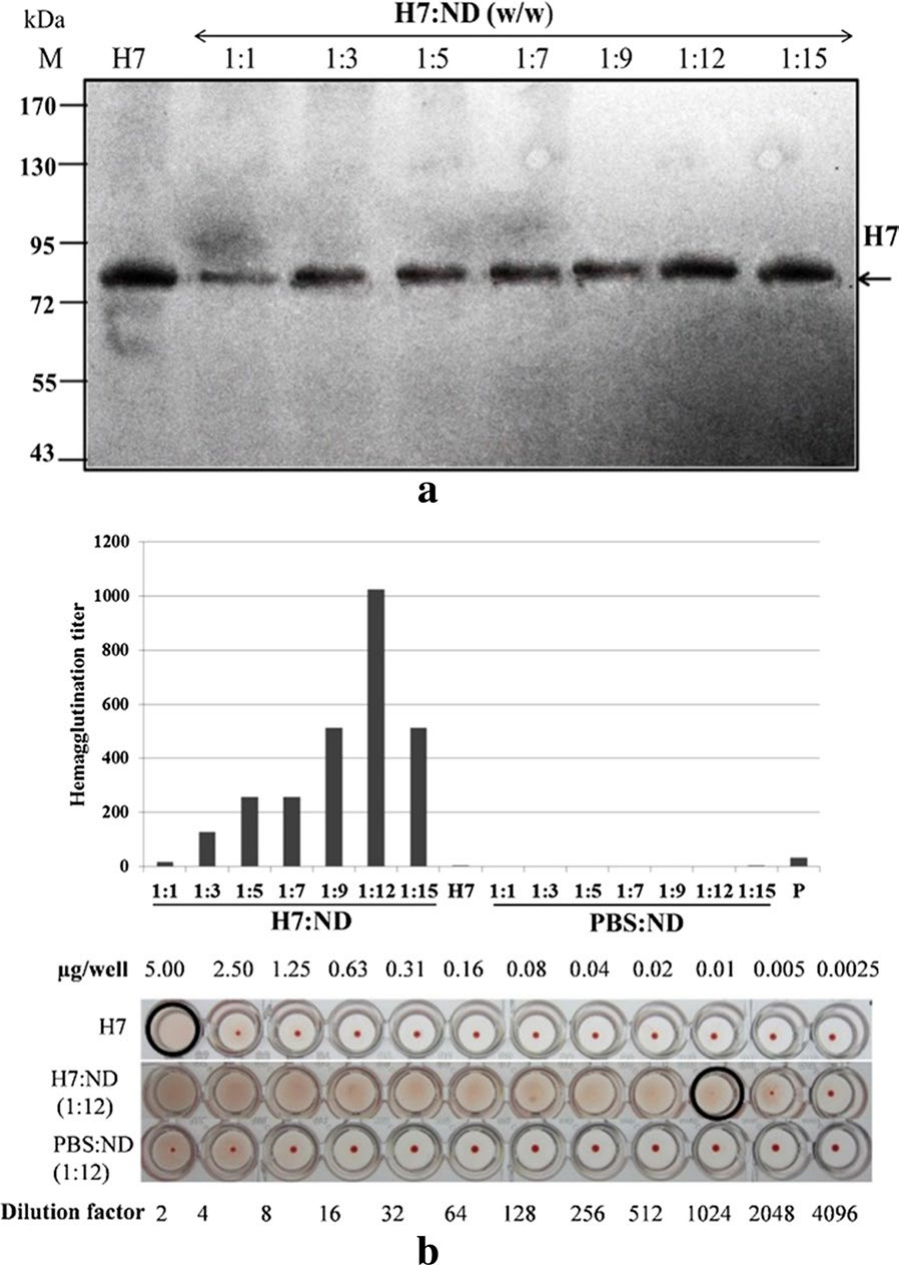
Western blot and bio-functional screening of different H7-ND conjugates. **a** Western blotting analysis confirms the conjugation of trimeric H7 with ND at different ratios. Trimeric H7 (100 ng/µL) was used for combining with NDs at different ratios (1:1, 1:3, 1:5, 1:7, 1:9, 1:12, 1:15, w/w). After mixing, the resulting products were washed in water and diluted in PBS. The same volume of H7 (2.5 µg) and all mixtures of H7:ND separated by 10% SDS-PAGE were blotted and detected with anti-c-myc monoclonal antibody followed by horseradish peroxidase-linked sheep anti-mouse IgG as a secondary antibody. **b** Bio-functional characterization of trimeric H7 and the mixture of trimeric H7 and ND via a haemagglutination assay. Trimeric H7 (100 ng/µL) was used for combining with NDs at different ratios (1:1, 1:3, 1:5, 1:7, 1:9, 1:12, 1:15, w/w). P: inactivated virus A/Hatay/2004/(H5N1) strain

To further prove the existence of trimeric H7 onto the surface of ND after protein coating, we analysed the H7:ND conjugates via SDS-PAGE and Western blot (Fig. 5a). Then, ImageJ was used to analyse the Western blot result based on the intensity of the H7 protein band. After the measurement, for the H7:ND ratio ranges of 1:1–1:12 (w/w), the binding capacity of trimeric H7 on the surface of NDs proportionally increased (from 20 to 82%) with the amount of NDs present in the H7–ND complexes. However, when adding more NDs that correspond to the H7:ND ratio of 1:15 (w/w), the binding capacity of trimeric H7 on the surface of NDs was not increased. This may explain that the binding capacity of trimeric H7 on the surface of NDs was saturated.

The biological activity of the purified trimeric Haemagglutinin proteins after mixing with NDs was assessed using a haemagglutination assay. The results of haemagglutination assay are shown in Fig. 5b. These results indicate that the ability to agglutinate chicken RBCs increased using the mixture of trimeric H7 and more ND, which corresponds to the H7:ND ratios of 1:1–1:12 (w/w). This confirms that H7:ND retained its function of RBCs binding ability. The mixture of H7:ND at the ratio of 1:12 (w/w) exhibited the highest haemagglutination activity (HAU = 1024 per 50 μL of the mixture of 100 ng/µL H7:1.2 µg/µL ND). In contrast, for the mixture with an H7:ND ratio of 1:15 (w/w), the HA titer was decreased (HAU = 512) due to an unknown reason. The PBS:ND at the ratio of 1:15 (w/w) (not other PBS:ND ratios) agglutinated chicken RBCs at HA titer of 4. This means that at a certain amount of ND, it can bind to receptors on the surface of RBCs to create the network with RBCs. We suggest a hypothesis that ND may act as a non-competitive/competitive inhibitor of H7 if too much NDs are present in the mixture of H7:ND (H7:ND ratio of 1:15, w/w). We observed that the free trimeric H7 (50 μL of 100 ng/µL = 5 µg) was able to agglutinate RBCs at HA titer of 2, which is 512-folds less than HA titer of the H7:ND mixture at a ratio (w/w) of 1:12. Therefore, these results indicate that the optimal H7:ND ratio (w/w) is 1:12.

### H7:ND elicited stronger H7 specific-IgG antibody responses than trimeric H7 shown by ELISA and Western blot

**Fig. 6:**
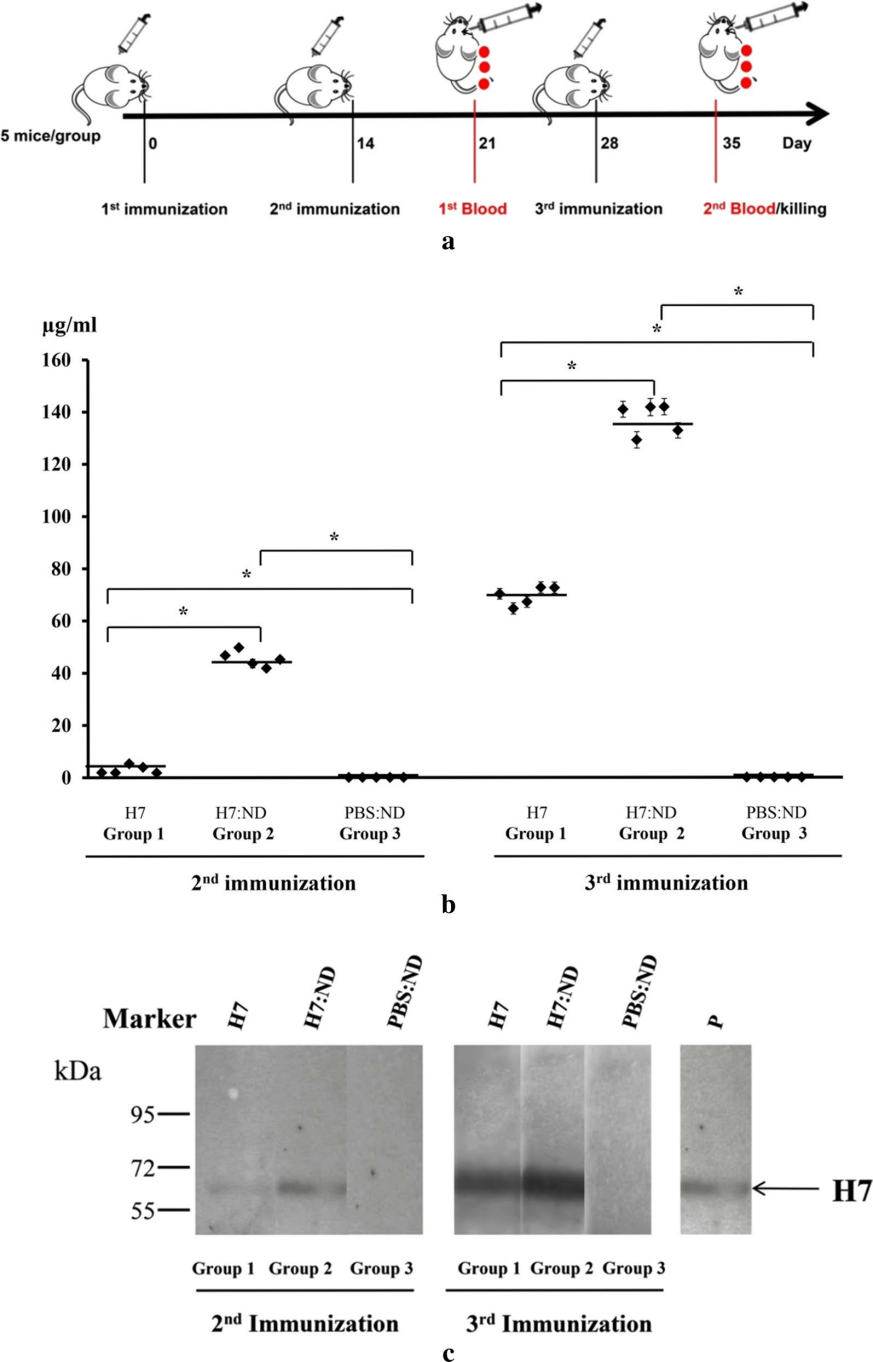
Immunopotentiation following immunization with an H7:ND (1/12, w/w) conjugate. **a** Immunization scheme in mice. **b** Measurement of H7 specific IgG amount in mouse sera via ELISA. In total, 50 ng of purified H7 [Influenza antigen A/Anhui/1/2013 (H7N9), NIBSC] per well were coated at the plate. The sera were diluted 1:5000 and a monoclonal mouse anti- H7N9 haemagglutinin/HA antibody (SinoBiological InC.) at concentrations of 0.5, 0.75, 1, 1.25, 2.5, 5, 12.5, 25, 50, 100, 150 μg/mL was applied as a standard, then analysed via ELISA. Specific immune responses were measured at 450 nm after 1 and 2 booster immunizations with H7 (group 1), H7:ND (group 2) and PBS:ND (group 3). The responses were recalculated according the standard values as µg/mL anti H7N9 antibody in the sera. The BSA background was subtracted. A standard curve was built by the help of OD450 values corresponding to known amounts of H7N9 haemagglutinin/HA antibody. The amount of H7 specific IgG antibody in mouse sera was measured via the standard curve. Statistical analyses were performed using the t-test (SigmaPlot) and are presented. A single dot indicates the value of a single mouse serum. SD was included on a single dot that corresponds to an ELISA data variation of a single mouse serum with three replications. The bars indicate the average value of the test groups. P < 0.05 was defined as a statistically significant difference. **c** Detection of H7-specific IgG antibodies via a Western blot. Sera from five mice from each group (against H7, H7:ND and PBS:ND as a negative control) were mixed, diluted 500 times and used as a primary antibody for detecting 100 ng of purified H7 [Influenza Antigen A/Anhui/1/2013 (H7N9), NIBSC]. Anti-mouse H7N9 haemagglutinin/HA antibody (SinoBiological InC.) was used as a primary antibody (positive control: P). HRP-linked goat anti-mouse IgG was used as a secondary antibody

Trimeric H7 (5 µg, group 1) and a mixture of trimeric H7 with NDs in a formulation containing 5 and 60 μg, respectively corresponds to the ratio of 1:12 (w/w, group 2) as well as ND (60 µg, group 3) separately diluted in PBS were used for immunization in mice (Fig. 6a). H7 specific IgG antibody responses in the sera of immunized mice were evaluated via ELISA and Western blot.

The measurement of H7 specific IgG amount in mouse sera via ELISA is shown in Fig. 6b. Each serum was measured in triplicate. All sera were diluted 1:5000. A monoclonal mouse anti- H7N9 haemagglutinin/HA antibody was applied as a standard. Specific immune responses were measured after 1 and 2 booster immunizations with H7 (group 1), H7:ND (group 2) and PBS:ND (group 3). The control values for BSA as an antigen at the solid phase in the ELISA were subtracted. The responses were recalculated according the standard values as µg/mL anti H7N9 antibody in the sera (see Materials and Methods). The mean values including the standard deviations as bars are represented in each dot. The square means for every group (5 individuals) were determined and compared by the t-test (Fig. 6b). The ELISA results showed that there was a statistically significant difference between mouse group 1 and group 2 (P < 0.05). Notably, after the 2nd and 3rd immunization, the ELISA results in Fig. 6b illustrate that H7:ND (group 2) induced higher amounts of H7 specific IgG antibodies in mouse sera (over 15.4- and 1.9-fold), respectively, than trimeric H7 did (group 1). In contrast, no H7 specific IgG antibodies were detected in mouse sera produced by immunization with PBS:ND (group 3).

Five mice sera collected after the 2nd and 3rd immunization of each group (H7:ND, H7 and PBS:ND) were mixed and used as a primary antibody for detecting purified H7. The Western blots in Fig. 6c indicate that stronger H7-specific IgG antibody responses were induced in mice vaccinated with H7:ND (group 2) than trimeric H7 (group 1). The H7 specific antibody responses were strongly increased after the 3rd immunization shown by Western blot analysis. In contrast, there was no H7 specific IgG antibody response induced in mice control group vaccinated with PBS:ND (group 3).

## Discussion

The H7-VLPs vaccine has been shown to elicit highly efficient and protective immune responses against the H7N9 influenza virus because of high molecular weight of H7 formation, which was formed by its self-assembly [9, 21, 23]. According to previous studies on the efficacy of neutralizing antibodies elicited by recombinant HA proteins from avian H5N1 influenza virus, a high-molecular-weight HA oligomer elicited the strongest neutralizing antibody response, followed by the HA trimer, while the monomer showed minimal efficacy [27]. For generating HA oligomers, it seems that recombinant HA fragments themselves cannot easily form functional, conformational trimeric or oligomeric structures, while the formation of these functional molecules of HA may be influenced by the addition of further sequences [3, 13, 20, 28].

In the present study, the trimeric H7 protein was formed by fusing H7 with a trimeric GCN4pII heptads repeat sequence derived from a wild-type dimeric GCN4 repeat found in *Saccharomyces cerevisiae* [7, 28]. To generate oligomeric H7 as a synthetic H7-VLP formation, trimeric H7 was physically mixed with a mixture of NDs in a wide range of sizes from ~ 50 to ~ 500 nm in diameter in optimized ratios. In the current study, we have shown that a facile and successful conjugation of H7 onto the surface of NDs can be obtained by simply mixing them together in DI-H_2_O. Characterization of the H7:ND complexes via size measurements, SDS-PAGE, Western-blot, and haemagglutination assay showed that recombinant proteins are non-covalently conjugated onto ND surface, and the activity of protein is well preserved after conjugation. In the previous study, it was shown that rH7 isolated from Sf9 cells represents high-molecular-weight VLP nanoparticles of approximately 20 nm that are likely comprised of 3–4 HA trimers, and that data suggest that the majority of H7 forms oligomeric VLP, which may be important for immunogenicity [23]. In the present study, the change in surface chemistry of NDs after trimeric H7 coating is not prominent since the Zeta-potential of ND and H7:ND was ~ − 45 and ~ − 38 mV, respectively. Since H7 is a glycoprotein, it is more likely that the protein does not interact with NDs through its glycan-expressing regions. After protein conjugation onto the ND surface, its glycans with negatively charged molecules are exposed to water. Because ND is a carbon-based nanoparticle, it likely interacts with the protein through hydrophobic forces as also observed for other membrane proteins [17]. Thus, the glycan expression domains of H7, which are mostly the epitopes of protein, are preserved and allow recognition by immune cells.

The optimal mixture of trimeric H7 and ND was further screened by Western blot and haemagglutination assay. Following removal of free proteins, antigen-loaded particles were recovered. Our data suggest that the mixture of H7:ND at a ratio of 1:12 (w/w) exhibited the highest binding capacity of trimeric H7 on the surface of NDs (82%), and agglutinated chicken RBCs at HA titer of 1024, which is 512-folds higher HA titer than its free trimeric H7. Notably, our synthetic H7-VLP exhibited a haemagglutination activity at a minimum total protein amount of 0.01 μg (Fig. 5b), which is lower than the minimum total protein amount of H7 VLP exhibiting haemagglutination activity from previous studies of Hu et al. [9] and Pushko et al. [23] of 0.098 and 0.039 μg, respectively. In contrast, in the present study, the free trimeric H7 agglutinated chicken RBCs at a minimum amount of total protein of 5 μg. These data suggest that ND presents a potential effect related to bio-function of H7 VLP shown in haemagglutination activity.

As expected, immunization with a physical mixture of H7:ND, which contains 5 μg of H7 and 60 μg of ND in formulation, elicited significantly higher H7-specific IgG antibodies over free trimeric H7 protein (shown in Western blot and ELISA result), which suggests a potential effect inherent to NDs. H7:ND may form oligomeric H7 as synthetic H7-VLPs that enhance H7 presentation and immune processing and are transported to draining lymph nodes for stronger H7-specific IgG immune responses. In a previous study, synthetic VLPs were prepared by mixing 100 nm gold nanoparticles and a spike protein [1]. Compared with inoculation with free proteins, vaccination with synthetic VLP showed enhanced lymphatic antigen delivery, stronger antibody titers, increased splenic T-cell response, and reduced infection-associated symptoms in an avian model of corona virus infection [1]. VLPs can act as adjuvants by carrying peptide sequences into antigen presenting cells and thus enhancing antigen processing pathways [25]. Surface-modified diamond nanoparticles can conformational stabilize proteins/peptides. NDs also provide a high degree of surface exposure to protein antigens. The elicitation of a strong and specific immune response as we observed can thus be explained by an adjuvant effect [15].

## Conclusions

In summary, synthetic H7-VLP was formed by mixing trimeric H7 antigen with synthetic NDs in optimized ratio and induced the assembly of a virus-like nanoparticle with viral trimeric H7 antigens covering the particulate core. Our result demonstrated the successful preparation of synthetic VLP via physical mixing of NDs with trimeric H7. Vaccination with the physical mixture of 5 μg of trimeric H7 protein and 60 μg of NDs elicited stronger H7 specific-IgG immune responses than free trimeric H7 protein. NDs have the potential to improve vaccine efficacy. NDs will continue to address challenges remaining in immunology and provide innovative strategies that can be broadly applied for the development of different vaccines in the future.

## Abbreviations

DI-H_2_O: deionized H_2_O
ER: endoplasmic reticulum
H7-pII (trimeric H7): H7 fused GCN4-pII motif protein
HA: haemagglutinin
HAU: haemagglutination unit
HRP: horseradish peroxidase
H7:ND: mixture of trimeric H7 and ND
IMAC: immobilized metal ion chromatography
ND: nanodiamond
RBCs: red blood cells
TEM: transmission electron microscopy
VLP: virus-like particles
